# Supplementary material for: Real Time Analysis of Bovine Viral Diarrhea Virus (BVDV) Infection and Its Dependence on Bovine CD46
Source: Viruses. 2020 Jan 17;12(1):116. doi: 10.3390/v12010116 (PMC7019258; doi:10.3390/v12010116)
Supplement: Supplementary file 1 [file viruses-12-00116-s001.zip › viruses-683406-SI.pdf]

Supplementary Figure 1

Localisation of virus particles on different parts of the cell surface at the start of the imaging period depending on the time after addition of $BVDV_{E2\text{-}mCherry}$ to SK6 $CD46_{fluo}$ (blue) or SK6 $CD46_{fluo}$ΔE2bind (green) cells in %.

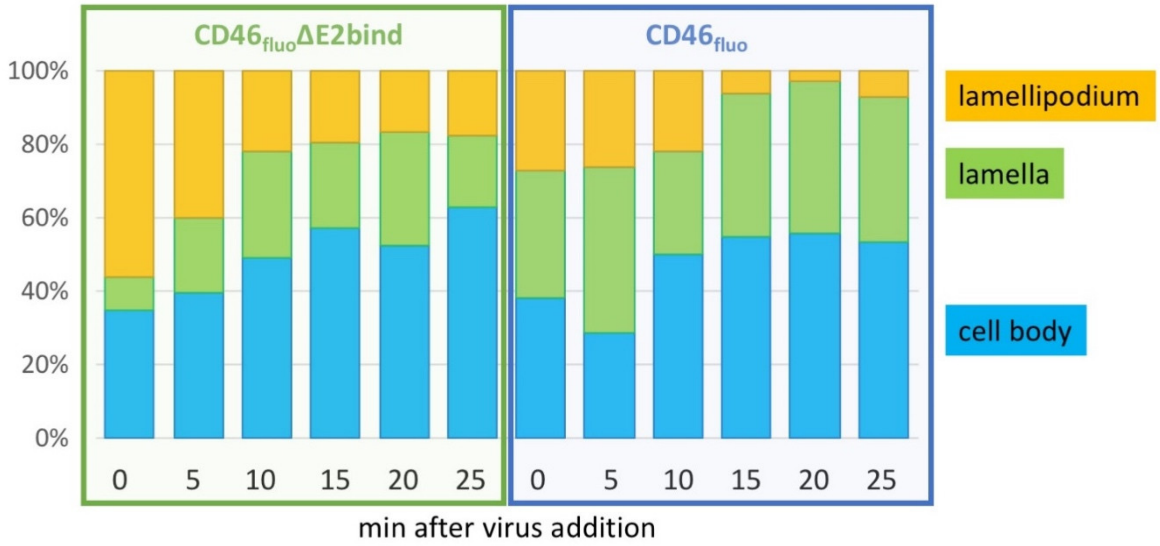

Supplementary figure 2

Directions of movements of virus particles on SK6 $CD46_{fluo}$ (blue) or SK6 $CD46_{fluo}\Delta E2bind$ (green) in %. Movement was judged with reference to the cell body, as indicated in the graph.

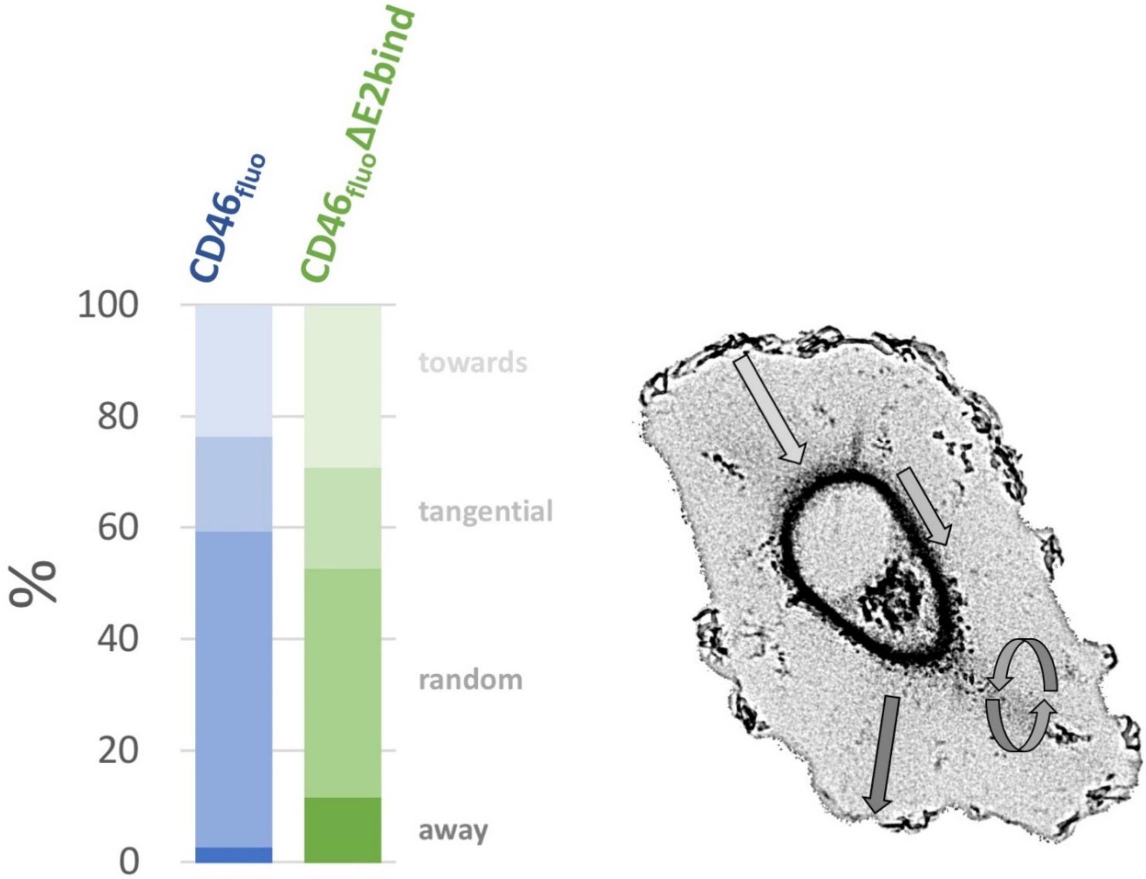

Supplementary Information

Script for the analysis of data generated with ImageJ’s manual tracking plugin.

```
#!/usr/bin/env python2
# -*- coding: utf-8 -*-
"""
Created on Tue Mar 26 08:38:17 2019

@author: christiane
"""

from itertools import groupby
import numpy as np
from sys import argv

script,infile = argv

a_file = np.loadtxt(infile, delimiter='\t', skiprows = 1, usecols = (0,1,2,3,4,5))

results = []
groups = []
for k, g in groupby(a_file, key=lambda x: x[0]):
   groups.append(list(g))

for g in groups:
   direct_distance = float((((int(g[0][2]))-(int(g[-1][2])))**2+((int(g[0][3]))-(int(g[-
1][3])))**2)**0.5)*0.13
   x = (float(i[4]) for i in g)
   velocities=[]
   previousLine=[]
   for i in g:
      if len(previousLine)==0:
         previousLine=i
      else:
         velocities.append(float(i[5])/float((int(i[1])-int(previousLine[1]))))
         previousLine=i
   stdv_velocity = float(np.std(velocities))
   min_speed = min(velocities)
   max_speed = max(velocities)
   real_distance = sum(x)+1
   mean_velocity = abs(real_distance / (int(g[0][1])-int(g[-1][1]))/10)
   directionality = direct_distance/real_distance
   length = int(g[-1][1]) - int(g[0][1])

print(g[0][0],direct_distance,real_distance,directionality,mean_velocity,min_speed,max_sp
eed, length)
```

```
results.append([g[0][0],direct_distance,real_distance,directionality,mean_velocity,stdv_velocity,min_speed,max_speed,length])
np.savetxt(infile+'_stats.txt', results , fmt = '%1.4f', delimiter='\t' , newline='\n' , header='track_ID\tdirect_distance\treal_distance\tdirectionality\tmean_speed\tstdv_speed\tmin_speed\tmax_speed\tlength')
```

Legends movie files:

Movie 1 and 2:

BVDV entry events. Single $BVDV_{E2\text{-}mCherry}$ particles (red, indicated by white arrow head) associated with the surface of SK TO $CD46_{fluo}$ cells ($CD46_{fluo}$ signal is shown in green) were imaged at a rate of one frame / 10s 15 or 25 min after the addition of virus, respectively. Scale bars represent 5µm and the time after start of the acquisition is shown in s in the upper left corner.

Movie 3:

Surfing of a $BVDV_{E2\text{-}mCherry}$ particle on the surface of a retraction fibre. Images were acquired at a rate of one frame / 10s. $BVDV_{E2\text{-}mCherry}$ signal is shown in red and the particle of interest is indicated by a white arrow head. $CD46_{fluo}$ signal is shown in green. For better visualization, two z-levels were merged in a maximum intensity projection. The time after start of acquisition is indicated in the upper left corner in seconds.

Movie 4 and 5:

Development of E2-mCherry (red) signal after infection of SK6 TO $CD46_{fluo}$ cells with an MOI of 1 (movie 4) or 10 (movie 5). Acquisition was started 60min after virus addition and data was acquired at a frame rate of one frame / 10min. $CD46_{fluo}$ signal is shown in green. The time after start of the acquisition is shown in the upper left corner in minutes.

Movie 6:

Colocalization of E2-mCherry and $CD46_{fluo}$ inside the cell. SK TO $CD46_{fluo}$ cells were imaged 20h after virus addition at a frame rate of one frame / 10s. $CD46_{fluo}$ signal is shown in green. The time after start of the acquisition is indicated in the upper left corner in seconds.

Sequence of the plasmid encoding the full-length genome of BVDV$_{E2\text{-}mCherry}$.

5' UTR: 1-384
Npro: 385-888
Core: 889-1194
Erns: 1195-1875
E1: 1876-2460
mCherry: 2461-3210
E2: 3211-4329
P7: 4330-4548
NS2: 4549-6429
NS3: 6430-8478
NS4A: 8479-9042
NS4B: 9043-9711
NS5A: 9712-11199
NS5B: 11200-13356
3'UTR: 13357-13582

GTATACGAGAACTAGATGAAATACTCGTATACATATTGGGCAATAAAAATTAATAATTAGGCCTAGGGAACAAATCCTCCTC
TGCGAAGGCCAAAAGGAGGCTAACCATGCCCTTAGTAGGACTAGCATAATGAGGGGGGTAGCAACAGTGGTGAGTTCGTT
GGATGGCTTAAGCCCTGAGTACAGGGCAGTCGTCAGTGGTTCGACGCCTTGGTTAAAAGGTCTCGAGATGCCACGTGGAC
GAGGGCATGCCCAAAGCACATCTTAACCTGAGCGGGGGTCGCTCAGGCGAAAACAGTTTAACCAACTGCTACGAATACAG
CCTGATAGGGTGCTGCAGAGGCCCACTGTATTGCTACTAAAAATCTCTGCTGTACATGGCACATGGAGTTGATTGCAAATG
AACTTTTATACAAAACATACAAACAAAAACCCGTTGGGGTGGAGGAACCTGTTTACGATCAGGCAGGTAATCCTTTATTTGG
TGAAAAGGGGGCAATCCACCCTCAATCGACGCTAAAGCTCCCACACAAGAGAGGGGAACGTGACGTTCCCATCAATTTGG
CATCTTTACCAAAAAGAGGTGATTGCAGGTCTGGTAACAGCAAAGGACCTGTGAGTGGAATCTACCTGAAGCCAGGGCCA
CTATTCTTTCAAGACTATAAGGGTCCCGTCTATCACAGAGCCCCACTGGAGCTTTTCGAGGAGGGATTTATGTGTGAAACA
ACTAAACGGATAGGGAGAGTAACTGGCAGTGACGGAAAGCTGTACCACATCTACGTGTGCATAGAAGGATGTATAACAGT
GAAGAGTGCCACAAGAAGTCACCAAAAGGTACTTAGGTGGGTCCACAACAGGCTCAACTGTCCTCTATGGGTCACAAGCT
GCTCAGATACAAAAGAAGAAGGGGCAACAAAAAAGAAACAACAAAAACCCGACAGACTGGAGAAGGGGAGGCTGAAAATA
GTGCCCAAAGAGTCTGAGAAAGACAGCAGGACTAAACCTCCGGATGCTACGATAGTGGTAGATGGGGTGAAATACCAGGT
AAAGAAGAAGGGGAAAGTCAAGAGCAAAAGCACACAGGACGGTTTATATCACAATAAAAATAAGCCGCCAGAATCACGCAA
GAAACTTGAGAAAGCATTATTGGCATGGGCAATATTGGCTATGGTCTTGATTCAAGTTACAATGGGTGAAAACATAACACAG
TGGAACCTACAAGATAATGGGACGGAAGGGATACAACGGGCAATGTTCCAAAGGGGGGTGAACAGAAGTCTACACGGCAT
CTGGCCAGAGAAAATCTGTACAGGTGTCCCTTCCCATCTAGCCACCGACGTGGAACTAAGAACAATCCATGGTATGATGGA
TGCAAGTGAGAAGACCAACTACACGTGTTGCAGACTTCAACGCCATGAGTGGAACAAACATGGTTGGTGCAACTGGTACA
ACATCGAACCTTGGATTTTAATCATGAATAGAACCCAAGCCAATCTTACTGAAGGTCAACCACTAAGAGAGTGCGCAGTCA
CTTGCAGATATGATAGGGATAGTGACCTAAATGTGGTAACACAAGCTAGAGATAGCCCCACGCCATTGACAGGCTGTAAGA
AAGGAAAAAATTTTTCTTTTGCAGGCATATTGATGCGGGGTCCCTGTAACTTTGAAATAGCTGCGAGTGATGTGTTGTTCAA
AGAACATGACTGCACAAGTGTGATTCAGGATACTGCTCATTACCTCGTAGACGGGATGACCAATTCCTTGGAAAGTGCCAG
ACAAGGGACCGCTAAACTGACAACCTGGTTAGGCAAGCAGCTACGGATACTTGGAAGGAAATTGGAAAACAAGAGCAAGA
CGTGGTTTGGAGCATATGCGGCTTCCCCTTACTGTGATGTTGATAAAAAACTTGGCTACATATGGTATACAAAAAATTGCAC
CCCTGCTTGCTTACCCAAGAACACAAAAATTATTGGCCCTGGGAAGTTTGATACTAACGCAGAGGATGGCAAGATATTGCA
TGAGATGGGAGGTCACTTGTCAGAGGTACTACTGCTTTCTTTAGTAGTGTTGTCTGATTTTGCACCAGAAACAGCTAGCGC
AACGTATCTAATTCTACATTTTTCCATCCCACAGAGCCATGTTGACATAATGGACTGTGATAAGACCCAATTGAACCTCACT
GTGGAGCTCACAACAGCAGATGTAATACCGGGGTCGGTCTGGAACCTAGGAAAATATGTCTGCATAAGACCAGATTGGTG
GCCTTATGAGACAGCTACAGTGCTGGCATTTGAAGAGGTGGGCCAGGTGATAAAGATAGTGCTGAGGGCACTTAGAGATT
TGACACGCATTTGGAACGCTGCCACGACCACAGCATTCTTAGTATGCCTTGTTAAGATGGTTAGGGGCCAGTTGGTACAAG
GCATCCTATGGCTGCTACTGATAACAGGGGTGCAAGGGCACGCGCGCATGGTGAGCAAGGGCGAGGAGGATAACATGGC
CATCATCAAGGAGTTCATGCGCTTCAAGGTGCACATGGAGGGCTCCGTGAACGGCCACGAGTTCGAGATCGAGGGCGAG
GGCGAGGGCCGCCCCTACGAGGGCACCCAGACCGCCAAGCTGAAGGTGACCAAGGGTGGCCCCCTGCCCTTCGCCTGG
GACATCCTGTCCCCTCAGTTCATGTACGGCTCCAAGGCCTACGTGAAGCACCCCGCCGACATCCCCGACTACTTGAAGCT
GTCCTTCCCCGAGGGCTTCAAGTGGGAGCGCGTGATGAACTTCGAGGACGGCGGCGTGGTGACCGTGACCCAGGACTC
CTCCCTGCAGGACGGCGAGTTCATCTACAAGGTGAAGCTGCGCGGCACCAACTTCCCCTCCGACGGCCCCGTAATGCAG
AAGAAGACCATGGGCTGGGAGGCCTCCTCCGAGCGGATGTACCCCGAGGACGGCGCCCTGAAGGGCGAGATCAAGCAG
AGGCTGAAGCTGAAGGACGGCGGCCACTACGACGCTGAGGTCAAGACCACCTACAAGGCCAAGAAGCCCGTGCAGCTGC
CCGGCGCCTACAACGTCAACATCAAGTTGGACATCACCTCCCACAACGAGGACTACACCATCGTGGAACAGTACGAACGC
GCCGAGGGCCGCCACTCCACCGGCGGCATGGACGAGCTGTACAAGCCATGGAAACATCATCATCATCATCATCATCATCT
AGACTGCAAACCTGAATACTCATATGCCATAGCCAAGAATGATAGAGTCGGCCTACTAGGAGCTGAAGGCCTTACCACTGT
TTGGGAGGAATACTCACCTGAAATGACGCTGGAAGACACAATGGTCATAGCCTCGTGCAGAGAAGGTAAGTTTACATACC
GCTCAAGGTGCACAAGGGAAGCTAGATATCTTGCAATTTTGCATTCAAGAGCCTTACCGACCAGTGTGGTATTTGAAAAAC
TTTTTGAGGGGCAAAAGCAAGAGGACACGGTCGAGATGGATGACAACTTCGAATTTGGACTCTGCCCATGCGACGCCAAG
CCCGTAGTAAAGGGGACTTTCAATACAACACTGCTAAATGGACCGGCTTTCCAGATGGTATGCCCCATAGGGTGGACAGG
GACCGTGAGCTGTATGTTAGCTAATAGGGATACCCTAGATACAGCAGTAGTGCGGACGTATAGGAGGTATAGACCATTCC
CTTACAGGCAAGACTGCATCACCCAAAAAGTTCTGGGGGAGGATCTCTATAACTGTATTCTTGGAGGAAACTGGACCTGTA
TAACTGGGGACCAACTACAATACTCAGGAGGCTCTATTGAATCCTGTAAGTGGTGTGGTTTTAAATTCCAAAGAAGTGAGG
GGTTACCACACTACCCCATTGGCAAGTGTAGGCTGAAGAATGAGACTGGCTACAGATTAGTAGACGACACCTCTTGCAATA
TAGGAGGTGTGGCGATAGTACCACAGGGGATGGTAAAGTGTAAGATAGGAGACACAATTGTACAGGTCATAGCTCTTGAC
ACCAAACTTGGGCCTATGCCCTGCAAGCCATATGAGATCATTTCAAGTGAGGGGCCCGTAGAAAAGACGGCATGTACCTT
CAACTACACGCGTACATTAAAGAATAAATACTTTGAGCCCAGAGACAGTTACTTCCAGCAATATATGCTAAAAGGAGAGTAT
CAATACTGGTTTGACCTGGAGGTCACTGACCATCACCGGGATTACTTCGCCGAGTCCATATTAGTGGTGGTGGTAGCTCTC
CTGGGTGGTAGATACGTGCTCTGGTTACTGGTCACATACATGGTCCTATCAGAACAAAAAGTCTTAGGGGCCCAATATGGG
ACAGGGACAGTGGTGATGATGGGCAACTTACTAACACATGATAGTGTTGAAGTGGTGACATATTTCTTGTTGCTATACCTAC
TGCTAAGAGAGGAGAACGTGAAGAAGTGGGTCTTACTCTTATACCATATCTTAGTGGCACACCCATTAAAATCAGTAACAGT
GATCTTGTTGATGATCGGGGATGTGGTGAAGGCTGATCCAGGGGACCAAGGGTACTTGGGGCAGATAGATGTCTGTTTCA

CAATGGTTGTACTGATCGTCATAGGTTTGATCATAGCCAGGCGTGACCCAACTATAGTGCCACTGGTCACAATAGTTGCAT
CACTGAGGGTCACTGGATTGACCTACGGACCTGGGTGGGATGTAGCTATGGCGGTCATGACCATAACCTTGCTAATGGTC
AGCTATGTGACAGATTACTTCAGATACAAAAGATGGCTGCAATGCATTCTCAGCCTTGTATCAGGGATGTTCTTGATAAGAA
GCCTAATACACCTAGGTAGAATTGAGATGCCAGGGGTGACCATCCCAAACTGGAGACCACTAACTTTAATTCTGCTATACC
TGATCTCAACAACAATTGTCACAATGTGGAAGGTTGACTTGGCTGGCCTATTGTTACAAGGTGTGCCTATCTTATTGCTGAT
CACAACCCTGTGGGCCGACTTCCTAACTCTTATACTGATTCTGCCCACCTACGAATTGGTTAAGTTATACTACCTGAAAACT
ATAAAGACTGATGTAGAAAGAAGCTGGCTAGGGGAGTTAGACTATAAAAGAGTTAACTCCGTCTACGATATTGATGAGAGT
GGAGAGGGCGTATATCTCTTTCCATCAAAGCAGAGGGCACAGAAAGACATCTCCATACTCTTGCCCCTTGTCAGAGCTACA
CTGATAAGTTGTGTTAGTAATAAATGGCAGCTAATATACATGGCTTACCTAACTGTGGACTTTATGTATTACATGCACAGGA
AGGTTATAGAAGAAATCTCAGGAGGCACCAACATGATATCCAGGTTAGTGGCAGCACTTATAGAGCTGAATTGGTCCATGG
AGGGAGAAGAAAGCAAAGGTCTGAAGAAGTTTTATTTATTGTCTGGAAGGTTAAGGAACCTAATAATAAAACACAAAGTGAG
GAATGATACCGTGGCTCGTTGGTACGGAGAGGAAGAAGTTTACGGCATGCCGAAGATCATGACTATAATAAAGGCTAGTA
CGCTGAATAAGAACAGGCACTGCATAATATGCACTGTATGTGAAGGCCGAAAATGGAAAGGTGGCACCTGCCCAAAATGC
GGACGCCATGGAAAACCCATAACATGTGGGATGTCACTAGCAGATTTTGAAGAAAGGCATTACAAAAGGATCTTTATAAGG
GAAGGCATTGTCAGCAACCCTGAAAGACGGAAGGAATATGAGATGAAACGAATGGCAGAAAATGAGCTGAGCCGGTCAGT
GAATGAGTTTCCGTCCAAGCTGCAAGAAGCAATGAATACGATGATGTGCAGCCGATGCCAGGGAAAGCATAGGAGGTTTG
AAATGGACCGGGAACCTAAGAGTGCCAGATACTGTGCTGAGTGTAATAGGCTGCATCCTGCTGAGGAAGGTGACTTTTGG
GCAGAGTCAAGCATGTTGGGCCTCAAAATCACCTACTTTGCGCTGATGGATGGAAAGGTGTATGATATCACAGAGTGGGC
TGGATGCCAGCGTGTGGGAATCTCCCCAGATACCCACAGAGTCCCTTATCACATCCCATTTGGTTCACGGATGCCAGGCA
CCAGTGGGCGGCAGAGAGCTACTCCAGATGCCCCTCCTGCTGACCTTCAGGATTTCTTGAGCCGGATCTTTCAAGTACCC
CCAGGCCAGATGTCCAACGGGAACTTCTTTGCAGCTCCTCAGCCCGGCCCTGGGGCCAACTTTGACGGCCCTTTCAGACA
AGAATATAATGGTTTTATACAATATACCGCTAGGGGGCAGTTATTTTTAAGAAACCTGCCCATACTGGCAACTAAAGTAAAA
ATGCTCATGGTAGGCAACCTCGGAGAAGAAATCGGGGACCTGGAACACCTTGGGTGGATCCTAAGGGGGCCTGCCGTGT
GCAAGAAGATCACAGAACATGAAAGATGCCACGTCAACATATTAGACAAGTTAACTGCATTTTTCGGGATCATGCCGAGGG
GGACTACACCCAGAGCTCCAGTGAGGTTCCCTACGAGTTTATTAAAAGTGAGGAGGGGTCTGGAGACTGGTTGGGCTTAT
ACACACCAAGGTGGGATAAGTTCGGTCGATCATGTAACCGCTGGCAAAGATCTATTGGTCTGTGACAGCATGGGGAGAAC
TAGAGTGGTCTGCCAGAGCAACAACAAGTTAACTGATGAGACAGAGTATGGTGTCAAGACTGATTCAGGATGCCCAGATG
GTGCCAGATGTTACGTGCTGAATCCAGAGGCAGTCAACATATCAGGGTCCAAAGGGGCAGTCGTCCACCTCCAAAAAACA
GGTGGGGAATTTACGTGTGTCACTGCATCAGGCACGCCGGCTTTCTTTGATCTAAAAAACTTGAAAGGATGGTCGGGCCT
GCCCATATTTGAAGCCTCCAGTGGAAGGGTAGTTGGCAGGGTTAAAGTAGGGAAGAATGAGGAGTCTAAACCTACAAAGA
TAATGAGTGGTATCCAGACCGTCTCAAAGAACACAGCAGATCTTACTGAAATGGTCAAGAAAATCACCAGTATGAACAGGG
GAGACTTCAAGCAGATAACTTTAGCAACAGGGGCAGGAAAAACTACAGAACTTCCAAAAGCGGTGATAGAGGAGATAGGA
AGACACAAGCGAGTGTTAGTCCTTATACCGTTGAGGGCAGCGGCAGAGTCAGTCTACCAGTACATGAGATTGAAACACCC
GAGTATATCCTTCAACCTAAGGATAGGGGACATGAAAGAAGGAGACATGGCAACAGGGATAACTTATGCATCATACGGGTA
CTTCTGCCAGATGCCTCAGCCAAAGCTTAGAGCAGCCATGGTAGAATACTCTTACATATTCTTAGATGAATACCATTGCGCC
ACTCCTGAACAACTGGCAATTATCGGAAAAATCCACAGATTTTCAGAGAGCATAAGAGTGGTTGCCATGACTGCCACCCCA
GCAGGGTCAGTAACCACAACGGGACAAAAACACCCAATAGAGGAATTCATAGCCCCCGAGGTGATGAAAGGGGAAGACCT
AGGCAGCCAATTCCTCGATATAGCGGGGCTAAAAATTCCCGTGGATGAGATGAAAGGCAACATGCTGGTTTTTGTACCCAC
GAGAAACATGGCAGTTGAGGTGGCGAAGAAGTTAAAAGCTAAGGGCTACAATTCTGGGTACTACTATAGTGGAGAGGATC
CAGCTAATCTGAGAGTTGTAACATCACAGTCCCCTTATGTAATTGTGGCAACGAACGCCATTGAATCAGGAGTGACATTAC
CAGACTTGGACACGGTCGTGGACACAGGGTTGAAATGTGAAAAGAGGGTGAGGGTATCATCAAAGATACCCTTCATCGTA
ACAGGCCTTAAGAGGATGGCCGTCACTGTGGGTGAACAGGCTCAACGTAGAGGCAGAGTAGGTAGAGTGAAGCCCGGAA
GATATTACAGGAGCCAGGAAACAGCAACCGGGTCGAAGGACTACCACTATGACCTCTTGCAGGCACAAAGATACGGCATT
GAGGACGGAATTAATGTAACAAAATCCTTTAGGGAAATGAATTATGATTGGAGCCTATACGAGGAGGACAGCCTGCTAATA
ACCCAGTTAGAAATATTAAATAATTTACTCATATCAGAAGACTTGCCAGCCGCTGTCAAGAACATAATGGCCAGGACTGATC
ACCCAGAGCCGATCCAACTTGCTTACAACAGTTATGAGGTCCAGGTCCCGGTCCTGTTCCCAAAAATAAGGAACGGAGAG
GTCACAGACACCTACGAAAACTACTCATTTCTAAACGCTAGAAAGTTAGGAGAGGATGTGCCCGTATACATCTATGCCACT
GAAGATGAGGACCTGGCAGTTGACCTCCTAGGGCTAGACTGGCCTGACCCTGGGAATCAGCAGGTCGTGGAGACCAGCA
AAGCACTGAAACAAGTGGCTGGGTTGTCTTCAGCTGAGAATGCCTTGCTCATAGCTTTATTTGGGTATGTAGGTTACCAAG
GTCTGTCAAAGAGGCATGTTCCAATGATCACAGATATATACACTATAGAGGACCAGAGACTAGAAGACACCACTCACCTCC
AGTATGCACCCAACGCCATAAAAACCGAGGGGACTGAGACTGAATTGAAGGAATTGGCGGCGGGTGACGTGGAAAAAATC
ATGGGAGCCATCTCGGATTATGCAGCTGGAGGACTGGAATTTGTAAAATCTCAAGCAGAAAAAGTGAAGACAGCCCCTTTG
TTTAAGGAAAACGTAGAAGCTGCAAAGGGGTACGTCCAAAAACTAATTGACTCATTAATTGAAGATAAAGATGTAATAATCA
GATATGGCTTGTGGGGAACACATACAGCACTCTATAAAAGCATAGCTGCAAGACTGGGGCATGAAACAGCATTTGCTACAC
TAGTAATAAAGTGGCTAGCCTTCGGAGGGGAGTCGGTGTCAGACCATGTCAGACAGGCGGCTGTTGATTTAGTGGTCTAT
TATGTGATGAACAAGCCTTCCTTCCCAGGTGATTCTGAAACCCAGCAAGAAGGGAGGCGGTTTGTCGCAAGCCTGTTCATC
TCCGCATTGGCAACTTACACATACAAAACCTGGAATTATCACAATCTCTCAAAAGTGGTGGAACCAGCCCTGGCATACCTC
CCTTATGCCACCAGCGCATTAAAAATGTTCACCCCAACGAGACTGGAAAGCGTGGTGATACTGAGCACTACAATATACAAA
ACATACCTCTCTATAAGGAAAGGGAAGAGTGATGGATTGCTGGGTACGGGGATCAGTGCAGCCATGGAGATCCTGTCACA
AAACCCGGCATCGGTAGGTATATCCGTGATGCTGGGGGTAGGTGCTATCGCTGCGCATAACGCCATTGAATCCAGCGAGC
AGAAAAGGACCCTACTTATGAAGGTGTTTGTAAAGAATTTCTTGGATCAGGCTGCAACGGATGAGCTGGTTAAAGAAAACC
CGGAAAAGATCATAATGGCTTTATTTGAAGCAGTCCAGACGATTGGCAACCCCTTAAGGTTGATATACCACCTGTATGGAG
TTTACTACAAGGGCTGGGAGGCCAAGGAGCTATCTGAGAGGACAGCAGGTAGAAACTTATTCACACTGATAATGTTCGAAG
CCTTCGAATTGTTAGGGATGGATTCAGAAGGGAAGATAAGGAACCTATCTGGAAACTACATCTTAGATTTGATCTATGGATT
GCATAGGCAGATCAACAGAGGGCTAAAGAAGATAGTACTGGGGTGGGCCCCTGCGCCCTTCAGCTGTGACTGGACTCCT
GGCGATGAGAGGATCAGGTTGCCAACGGACAACTATTTAAGAGTGGAAACCAGGTGCCCGTGTGGCTATCAGATGAAAGC
TATCAAAAATGTAGATGGTAAGCTTAACAAGGTGGAGGAGAGAGGACCTTTTCTATGTAGGAACAGACCTGGTAGGGGGC
CAGTCAACTACAGAGTTACCAAGTACTATGATGACAATCTCAAAGAGATAAAGCCAGTGGCAAAGTTAGAAGGACAGGTGG
AGCACTACTACAAAGGGGTCACGGCAAAAATTGACTACAGTAAAGGGAAAATGCTTTTGGCTACTGATAAATGGGAGGTGG
AACATGGTACAATAACCAGGTTGGCTAAGAGATATACTGGGGTCGGGTTCAATGGCGCATACCTAGGCGACCAGCCCAAC
CACCGTGACCTAGTGGAGAGGGATTGTGCAACTATAACAAAAAACACAGTGCAGTTTCTAAAGATGAAGAAGGGGTGTGC
ATTTACCTATGATCTAACCATCTCCAATTTGACCAGGCTCATCGAACTAGTTCACAGAAACAACCTTGAAGAGAAGGAAATA
CCCACCGCCACGGTCACTACGTGGCTAGCTTACACCTTCGTGAACGTAGACGTGGGGACCATAAAACCGGTGCTAGGGG
AGAGAGTAATCCCCGATCCTGTAGTTGATGTCAACTTACAACCAGAGATCCAAGTGGATACATCAGAGGTTGGGATCACAA
TAATTGGAGGGGGAGCCTTGATGACAACAGGAGTGACACCCGTTGTGGAAAAGATAGAGCCTGATGCTGACGGCAACCAA

AACTCGGTGAAGGTCGGGCTGGATGAAGGTAATTATCCAGGGCCTGGTGTACAAACACACACACTAGTAGAAGAAATACA
CAACAGGGACGCAAGACCCCTCATTCTGGTCCTAGGCTCAAAGAGTTCCATGTCAAATAGAGCAAAGACAGCTAAAAATAT
AAACCTGTATACAGGAGATGACCCCAGGGAGATAAGAGACTTGATGGCAGAAGGACGCTTGTTAGTAGTGGCATTGAGGC
ACATCGACCCTGATCTACTTGAACTAGTTGACTTCAAGGGGACCTTTTTAGATAGGGAAACCTTGGAGGCTTTGAGTCTTG
GGCAGCCCAGACCCAAGCGGGTTACCAAAGCAGTAATTAGGGAATTATTGAAAGAGGAAAGGCAAGTGGAGATCCCTAAC
TGGTTTACATCAGATGATCCGGTATTCTTGGAAGTAGCCATGAATAAAAATAAGTACCACTTAGTGGGAGATGTAGGAGAG
GTGAAAGACCGAGCTAAGGCACTTGGGGCTACGGATCAGACAAGAATAATAAAGGAGGTAGGCTCAAGGACATATACCAT
GAAGTTGTCTAGCTGGTTCCTACAAGCGTCAAACAAACAGATGAGTCTAACTCCACTGTTTGAGGAACTGCTGCTACGATG
CCCCCCTGCAACTAAGAGCAATAAGGGACATATGGCATCAGCTTACCAATTGGCACAGGGCAATTGGGAGCCCCTCGGTT
GTGGGGTGCATCTAGGTACCATACCAGCCAGAAGAGTGAAGATACATCCATACGAAGCTTATCTGAAGCTGAAAGACCTC
GTAGAAGAAGAGGAAAAGAAGCCAAGGATTAGGGATACAGTAATAAGGGAGCACAACAAATGGATACTTAGAAAAATAAAA
TTCCAAGGAGACCTCAAGACTAAGAAAATGCTCAACCCTGGAAAACTATCTGAACAGCTGGACAGAGAGGGGCACAAAAG
AAACATCTATAATAACCAGATCAGTACCATAATGTCTAGTGCAGGCATACGGCTGGAAAAGTTGCCAATAGTGAGGGCCCA
AACCGACACTAAGAGCTTCCATGAGGCAATAAGGGATAAGATAGACAAGAATGAGAACCGGCAAAATCCAGAATTGCACAA
CAAATTGTTGGAAATTTTTTACACAATAGCCAACCCCACCCTACAACACACCTACGGTGAGGTAACGTGGGAGCAACTTGA
GGCAGGAATAAATAGGAAAGGGGCAGCAGGCTTCCTAGAGAAGAAGAACATCGGGGAAGTATTGGAATCAGAAAAACACC
TGGTAGAACAATTGATCAGGGATATGAAGGCCGGGAGAAAGATAAGGTATTATGAAACAGCAATACCAAAAAATGAGAAGA
GAGATGTTAGTGACGACTGGCAAGCCGGGGACCTGGTAGATGAGAAGAAGCCAAGAGTCATCCAATACCCTGAAGCCAA
GACAAGGTTAGCCATCACTAAGGTCATGTATAACTGGGTAAAACAGCAACCCGTTGTGATCCCAGGATACGAAGGGAAGA
CCCCCTTGTTCAACATCTTTAATAAAGTGAGGAAGGAATGGGACTTGTTCAATGAGCCAGTGGCCGTAAGTTTTGATACCA
AAGCTTGGGATACCCAAGTGACTAGTAGGGATCTACAACTTATTGGAGACATCCAGAAATACTACTACAGGAAGGAATGGC
ACAAGTTCATTGACACCATCACTGACCACATGGTAGAAGTGCCAGTAATAACAGCTGATGGTGAAGTATATATAAGAAATG
GGCAGAGGGGGAGTGGCCAGCCAGACACAAGTGCAGGCAACAGTATGTTAAATGTCCTGACAATGATGTATGCTTTCTGC
GAAAGCACAGGGGTCCCGTACAAGAGTTTCAACAGGGTGGCAAGGATCCATGTCTGTGGGGACGATGGCTTCTTAATAAC
TGAAAAAGGCTTAGGGTTAAAATTTGCCAACAAGGGGATGCAAATCCTTCACGAAGCAGGCAAGCCCCAGAAAATAACGG
AAGGGGAAAAAATGAAAGTTGCCTATAGATTTGAAGACATAGAATTTTGCTCACATACCCCAGTCCCTGTTAGGTGGTCTGA
TAACACCAGTAGTTACATGGCCGGCAGAGACACTGCCGTGATACTATCAAAGATGGCAACAAGATTGGATTCAAGCGGGG
AGAGGGGTACCACGGCATGTGAAAAAGCAGTGGCTTTCAGCTTCTTACTAATGTACTCCTGGAACCCGCTTGTTAGGAGG
ATTTGCCTATTGGTCCTTTCGCAGCGACCAGAAATAGCTCCATCAACACAGACCACTTATTACTACAAAGGAGATCCAATAG
GGGCCTATAAAGATGTAATAGGCCGGAATCTAAGTGAACTAAAGAGAACAGGCTTCGAGAAATTGGCAAATCTAAATCTGA
GTCTGTCCACGCTAGGGATCTGGACCAAACATACAAGCAAAAGAATAATTCAGGACTGCGTGGCCATTGGAAAGGAAGAA
GGAAATTGGCTGGTAAATGCCGACAGGTTGATATCCAGCAAAACTGGCTACTTATACATACCTGACAAAGGTTTTACAATAC
AAGGAAAGCATTATGAGCAACTGCAACTAGGAGCGCGCACGAACCCGGTTATGGGTGTCGGGACTGAGAGGTACAAGTTA
GGTCCCATAGTCAATCTGCTATTGAGAAGGTTGAAGGTCCTGCTCATGGCGGCTGTCGGCGCCAGCGGCTGAAACAAATG
TATATACTATAAATAAATTGACCCTTGTACATATTGTATATAAATATAGTTGGGATCGTCTACCTGAAAAAGACAATACACCC
AACATTAACAGCTAAATAGTGGTTGAGATTATCTACCTCAAGATAACACTACACTCAATGCACATAGCACTTTAGCTGTATGA
GGATATGCCCGACGTCTACAGTTGGACTAGGGAAGACCTCTAACAGCCCGGGCGGATCCTTGGATTAAGTACCTCACTAA
CGTTCCAAAAGGGAACTTTACAGCCACTTTAGAAGAATGGCAGGCGGAACACGAGGACATTATGAAGGCCATTAATTCTAC
ATCCACAGTATCTGACCCTTTCGCCAGCAAAGTGAATACATGCTGGGCTAAAGCTATTATACCCATCCTAAGAACGGCAGG
GATAGAACTTACATTCGAGCAGTGGGAAGATCGCTTATCGCGATACCGTCGAGGGGAATTAATTCTTGAAGACGAAAGGG
CCAGGTGGCACTTTTCGGGGAAATGTGCGCGGAACCCCTATTTGTTTATTTTTCTAAATACATTCAAATATGTATCCGCTCA
TGAGACAATAACCCTGATAAATGCTTCAATAATATTGAAAAAGGAAGAGTATGAGTATTCAACATTTCCGTGTCGCCCTTATT
CCCTTTTTTGCGGCATTTTGCCTTCCTGTTTTTGCTCACCCAGAAACGCTGGTGAAAGTAAAAGATGCTGAAGATCAGTTGG
GTGCACGAGTGGGTTACATCGAACTGGATCTCAACAGCGGTAAGATCCTTGAGAGTTTTCGCCCCGAAGAACGTTTTCCAA
TGATGAGCACTTTTAAAGTTCTGCTATGTGGCGCGGTATTATCCCGTGTTGACGCCGGGCAAGAGCAACTCGGTCGCCGC
ATACACTATTCTCAGAATGACTTGGTTGAGTACTCACCAGTCACAGAAAAGCATCTTACGGACACCAGTCACAGAAAAGCAT
CTTACGGATGGCATGACAGTAAGAGAATTATGCAGTGCTGCCATAACCATGAGTGATAACACTGCGGCCAACTTACTTCTG
ACAACGATCGGAGGACCGAAGGAGCTAACCGCTTTTTTGCACAACATGGGGGATCATGTAACTCGCCTTGATCGTTGGGA
ACCGGAGCTGAATGAAGCCATACCAAACGACGAGCGTGACACCACGATGCGTGCAGCAATGGCAACAACGTTGCGCAAA
CTATTAACTGGCGAACTACTTACTCTAGCTTCCCGGCAACAATTAATAGACTGGATGGAGGCGGATAAAGTTGCAGGACCA
CTTCTGCGCTCGGCCCTTCCGGCTGGCTGGTTTATTGCTGATAAATCTGGAGCCGGTGAGCGTGGGTCTCGCGGTATCAT
TGCAGCACTGGGGCCAGATGGTAAGCCCTCCCGTATCGTAGTTATCTACACGACGGGGAGTCAGGCAACTATGGATGAAC
GAAATAGACAGATCGCTGAGATAGGTGCCTCACTGATTAAGCATTGGTAACTGTCAGACCAAGTTTACTCATATATACTTTA
GATTGATTTAAAACTTCATTTTTAATTTAAAAGGATCTAGATCCTTTTTGATAATCTCATGACCAAAATCCCTTAACGTGAGTT
TTCGTTCCACTGAGCGTCAGACCCCGTAGAAAAGATCAAAGGATCTTCTTGAGATCCTTTTTTTCTGCGCGTAATCTGCTGC
TTGCAAACAAAAAAACCACCGCTACCAGCGGTGGTTTGTTTGCCGGATCAAGAGCTACCAACTCTTTTTCCGAAGGTAACT
GGCTTCAGCAGAGCGCAGATACCAAATACTGTCCTTCTAGTGTAGCCGTAGTTAGGCCACCACTTCAAGAACTCTGTAGCA
CCGCCTACATACCTCGCTCTGCTAATCCTGTTACCAGTGGCTGCTGCCAGTGGCGATAAGTCGTGTCTTACCGGGTTGGA
CTCAAGACGATAGTTACCGGATAAGGCGCAGCGGTCGGGCTGAACGGGGGGTTCGTGCACACAGCCCAGCTTGGAGCGA
ACGACCTACACCGAACTGAGATACCTACAGCGTGAGCTATGAGAAAGCGCCACGCTTCCCGAAGGGAGAAAGGCGGACA
GGTATCCGGTAAGCGGCAGGGTCGGAACAGGAGAGCGCACGAGGGAGCTTCCAGGGGGAAACGCCTGGTATCTTTATAG
TCCTGTCGGGTTTCGCCACCTCTGACTTGAGCGTCGATTTTTGTGATGCTCGTCAGGGGGGCGGAGCCTATGGAAAAACG
CCAGCAACGCGGCCTTTTTACGGTTCCTGGCCTTTTGCTGGCCTTTTGCTCACATGTTCTTTCCTGCGTTATCCCCTGATTC
TGTGGATAACCGTATTACCGCCTTTGAGTGAGCTGATACCGCTCGCCGCAGCCGAACGACCGAGCGCAGCGAGTCAGTG
AGCGAGGAAGCGGAAGAGCGCCTGATGCGGTATTTTCTCCTTACGCATCTGTGCGGTATTTCACACCGCATATGGTGCAC
TCTCAGTACAATCTGCTCTGATGCCGCATAGTTAAGCCAGTATACACTCCGCTATCGCTACGTGACTGGGTCATGGCTGCG
CCCCGACACCCGCCAACACCCGCTGACGCGCCCTGACGGGCTTGTCTGCTCCCGGCATCCGCTTACAGACAAGCTGTGA
CCGTCTCCGGGAGCTGCATGTGTCAGAGGTTTTCACCGTCATCACCGAAACGCGCGAGGCAGCTGCGGTAAAGCTCATCA
GCGTGGTCGTGAAGCGATTCACAGATGTCTGCCTGTTCATCCGCGTCCAGCTCGTTGAGTTTCTCCAGAAGCGTTAATGTC
TGGCTTCTGATAAAGCGGGCCATGTTAAGGGCGGTTTTTTCCTGTTTGGTCACTGATGCCTCCGTGTAAGGGGGATTTCTG
TTCATGGGGGTAATGATACCGATGAAACGAGAGAGGATGCTCACGATACGGGTTACTGATGATGAACATGCCCGGTTACT
GGAACGTTGTGAGGGTAAACAACTGGCGGTATGGATGGCGGCCGCGATTTAGGTGACACTATA
